# Fibrous Ankylosis

**Published:** 1892-08

**Authors:** J. A. Nunn

**Affiliations:** Principal Lahore Veterinary College; and Veterinary-Lieutenant Blenkinsop A.V.D., Veterinary-Surgeon to the Punjab Government


### SELECTIONS.

### FIBROUS ANKYLOSIS.

By Veterinary-Captain J. A. Nunn, D.S.O., F.R.C.V.S.

*Principal Lahore Veterinary College; and Veterinary-Lieutenant Blenkinsop A.V.D., Veterinary-Surgeon to the Punjab Government.*

It is very common, in equine patients, to find that certain joints have, more or less, lost their power of flexion, owing to inflammatory processes of various kinds in and around them, the exciting causes being injuries or overwork. This stiffness or loss of flexion is most commonly seen in the front fetlock joints, but the writers of this article have seen cases in which the pastern, knee, and hind fetlock joints have been affected. In cases where this loss of flexion is due to an inflammatory exudate having been thrown out and become organized, good results have been obtained from placing the patient under chloroform, and breaking down these adhesions by forcible extension and flexion of the affected joint. This process is the same as that carried out from time immemorial under the name of "bone-setting," and was formerly chiefly confined to empirics. These men were often very expert in the manipulation of such joints, both in human beings and animals; and amongst laymen they had a great reputation, and received a considerable amount of credit for what was supposed to be their superior anatomical knowledge; whereas their views, so far as fractures and dislocations were concerned, were often perfectly erroneous. The subject has been taken up and carefully studied during the last few years by human surgeons, and it was from information published from time to time that we were first induced to try this line fo treatment in veterinary practice. In the *British Medical Journal* for 26th May, 1888, a valuable paper, read by Mr. W. J. Penney, F.R.C.S., before the Somerset branch of the British Medical Association, is published, and from this paper a great deal of what is now written has been drawn. Mr. Penney states:

"Among affections successfully treated in this way are cases of fibrous adhesions within and around the joints, ligaments and synovial membranes; cases of true, complete dislocation; cases of partial dislocation or subluxation; cases of adhesions of tendons to each other, or to ligaments or other structures; disarrangement of ligaments; displacement of tendons; cases of muscular spasm; ganglia which have been ruptured. In some cases bodies foreign to the part, such as new growths, detached pieces of cartilage, displaced cartilages, thickened synovial fringes, organized blood-clot, and numerous others, have become nipped between the bones forming a joint, and have thus interfered with its function." Now veterinary surgeons will at once notice how frequently cases of lameness in horses are attributable to one or several of these causes, and they will be able to call to mind numerous cases which have, for hardly any noticeable cause, been lame for lengthy periods. We have ourselves had several cases which have been under treatment for months, and which had been blistered over and over again, and, in some instances, fired, brought to us as being incurably lame; and these cases, after the affected part had been manipulated once or twice under chloroform, have been able to trot fairly sound, and perform useful work.

The chief structures in which lesions occur, causing loss of power of flexion, are the capsular and synovial ligaments; and in the term capsular ligament, as here used, are included the lateral ligaments with the anterior and posterior ligaments of the same joint. The tendons are less frequently implicated, but in the fetlock joint the branches of the superior sesamoidal ligament are very commonly included in the tissues which have become matted together. The majority of adhesions of this sort take place after strains, and are less frequently the result of sub-luxations.

Lameness is chiefly caused by the capsule becoming pinched between the bones forming the joint, owing to its becoming sticky and thickened from the fibrinous effusion on it, and consequently not being able to be drawn out of the way, or to fold in a natural manner, so as not to interfere with the movements of the joint. As long as the joint is worked, possibly the pain on movement will be shown, and it is only after the adhesions have been broken down that the joint can be extended and flexed with immunity from pain.

With regard to the selection of cases suitable for manipulation, the following are the chief symptoms to be noted: Total absence of any acute inflammatory symptoms, shown by the absence of heat in the joint; loss of power of flexion of the joint, which can be ascertained by lifting the joint and comparing the extent it can be flexed with that of the corresponding healthy one. In making this comparison, care should be taken that the force be applied to the toe of the foot, and that exactly the same amount be applied to both limbs. To gauge the amount of force necessary to produce corresponding flexion in both limbs, an ordinary spring balance, such as is used by butchers, can be employed, the hook being fixed between the toe of the shoe and the sole. This flexion sets up muscular spasms of a varying degree, that in the fore limbs, when the knee or fetlock joints are affected, are chiefly located in the muscles of the fore-arm and elbows. On gentle main pulsation, the movement of the joint should be perfectly smooth, and there should be but little thickening round it. If there is much thickening, it is advisable to reduce it as much as possible by gentle friction with some absorbent agent, and exercise, and the following ointment has been found very useful for the purpose:

R. Unguent. Hydrarg. Fort., 1 dram.

Vaseline, 1 oz.—1½ oz.

(Misce.)

Having decided to manipulate the joint, the patient should be cast and placed completely under the influence of chloroform. It is most important that perfect anesthesia is produced in the larger animals, for if this is not done, the muscular spasm is so powerful than when any attempt is made to flex the joint, either it cannot be overcome, or else, if it is, such force has to be applied that there is great risk of a worse injury than that already existing taking place. The limb should be removed from the hobbles and the joint extended and flexed several times. The amount of force necessary varies with the extent and age of the adhesions; but force should not be spared until the joint has been fully flexed and extended. It is also important that a steady and increasing pressure be used, not sudden jerks. The adhesions usually give way with a sharp snap or crack, and from this it was thought that the bone-setters reduced dislocations. In the case of a stiff knee or fetlock joint, a piece of webbing should be passed round the foot, in order to obtain sufficient power to extend and flex the joint. The following method of fastening on this webbing is what is recommended: A piece of webbing or cotton belting about one inch wide and ten feet long is required. In the centre of this a clove hitch is made.

The free ends of this webbing are twisted round each other twice, and are then carried forward *over* the hind and *under* the fore part of the clove hitch, then backwards and *over* the fore part of the clove hitch, and again *forward* and *under*, so as to lap once round the fore part of it. The web is now adjusted round the foot as follows: The clove hitch is placed round the foot with the twists formed at the heel. The ends of the webbing are underneath the foot, and extend from the cleft of the frog to the toe. The ends are in front of the toe, and by pulling on the loose ends the whole webbing is tightened, and it will be found that great force can be exerted on the fetlock-joint without the webbing slipping off.

The after-treatment consists of dry massage, with flexion and extension of the joint for half an hour to an hour morning and evening. And if this treatment is properly carried out, it will be found that there is little or no effusion resulting from the violent manipulation the joint has received. In some cases it is necessary to flex the joint more than once before its natural extent of movement returns, and till this result is obtained the treatment should be persevered with, the horse being cast every seven or eight days, and the joint manipulated under chloroform. Six cases have been treated by this method at the Lahore Veterinary College during the past winter session, all of them with good results. We would suggest that this method of extension might be tried in the numerous cases of thickened and stiffened joints in the hunters that are seen at the end of the hunting season, before the usual remedy of the actual cautery is applied. Of course, if counter-extension fails, the cautery can be used afterwards; but it appears worthy of a trial, if only to avoid the blemish caused by firing.—*The Veterinary Journal.*

